# Long term monitoring of jaguars in the Cockscomb Basin Wildlife Sanctuary, Belize; Implications for camera trap studies of carnivores

**DOI:** 10.1371/journal.pone.0179505

**Published:** 2017-06-28

**Authors:** Bart J. Harmsen, Rebecca J. Foster, Emma Sanchez, Carmina E. Gutierrez-González, Scott C. Silver, Linde E. T. Ostro, Marcella J. Kelly, Elma Kay, Howard Quigley

**Affiliations:** 1 Environmental Research Institute, University of Belize, Belmopan, Belize; 2 Panthera, 8 West 40th Street, 18th Floor, New York, NY, United States of America; 3 Facultad de Ciencias Naturales, Universidad Autonoma de Queretaro, Querétaro, Querétaro, México; 4 Wildlife Conservation Society, 2300 Southern Boulevard, Bronx, NY, United States of America; 5 New York Academy of Sciences, 7 World Trade Center, New York, NY, United States of America; 6 Department of Fisheries and Wildlife Sciences, Virginia Tech, Blacksburg, VA, United States of America; University of Alberta, CANADA

## Abstract

In this study, we estimate life history parameters and abundance for a protected jaguar population using camera-trap data from a 14-year monitoring program (2002–2015) in Belize, Central America. We investigated the dynamics of this jaguar population using 3,075 detection events of 105 individual adult jaguars. Using robust design open population models, we estimated apparent survival and temporary emigration and investigated individual heterogeneity in detection rates across years. Survival probability was high and constant among the years for both sexes (φ = 0.78), and the maximum (conservative) age recorded was 14 years. Temporary emigration rate for the population was random, but constant through time at 0.20 per year. Detection probability varied between sexes, and among years and individuals. Heterogeneity in detection took the form of a dichotomy for males: those with consistently high detection rates, and those with low, sporadic detection rates, suggesting a relatively stable population of ‘residents’ consistently present and a fluctuating layer of ‘transients’. Female detection was always low and sporadic. On average, twice as many males than females were detected per survey, and individual detection rates were significantly higher for males. We attribute sex-based differences in detection to biases resulting from social variation in trail-walking behaviour. The number of individual females detected increased when the survey period was extended from 3 months to a full year. Due to the low detection rates of females and the variable ‘transient’ male subpopulation, annual abundance estimates based on 3-month surveys had low precision. To estimate survival and monitor population changes in elusive, wide-ranging, low-density species, we recommend repeated surveys over multiple years; and suggest that continuous monitoring over multiple years yields even further insight into population dynamics of elusive predator populations.

## Introduction

Managing mammals of conservation concern requires accurate and precise estimates of population size through time and across space. Population estimation of elusive, wide-ranging species, living at low density, for example large forest-dwelling carnivores, is particularly difficult due to their low probability of detection. Camera traps have become a popular tool for providing capture-recapture records for estimating population size (abundance or density) of many such carnivore species, particularly those with individually unique coat patterns [1, 2, 3]. Generic monitoring protocols have been developed and implemented for a range of different species (e.g. tigers, *Panthera tigris*, [4]; leopards, *Panthera pardus*, [5]; jaguars, *Panthera onca*, [6, 7]). Many researchers have used camera traps for one-off ‘closed’ population estimates from single surveys, providing a ‘snap shot’ of the population status (for examples see [3]). Others have now been using camera traps at the same sites over multiple years for estimating life history parameters and assessing population dynamics (e.g. tigers, [8]; snow leopards, *Panthera uncia*, [9]; jaguars, [10]). In this study, we estimate life history parameters and abundance for a protected jaguar population using camera-trap data from a 14-year monitoring program (2002–2015) in Belize, Central America.

Long-term studies following individuals of a population through time are rare [11] yet necessary for detecting and responding to population change in species of conservation concern or management interest. For example, monitoring the effectiveness of a protected area, ensuring hunting is sustainable, and/or managing human-carnivore conflict, are all relevant to the management of large felids. In the case of jaguars, monitoring populations through time is relevant due to their range contraction and near-threatened status [12, 13], the illegal trade in their body parts, and their important ecological and economic roles as an umbrella species, a charismatic species of value to the eco-tourism industry, and as a species that is persecuted in retaliation for livestock depredation [14, 15, 16, 17, 18].

Life history parameters (e.g. survival/mortality, recruitment, longevity, sex ratio) obtained from long-term monitoring of predators are used widely for assessing population viability and predicting population change under alternative scenarios (e.g. habitat fragmentation, [19, 20]; disease, [21], climate change, [22]; and poaching/harvesting/ trophy hunting, [23, 24, 25]. These parameters are relatively well-known for a number of large felids (e.g. tigers [8, 26, 27]; leopards [28, 29, 30]; lions, *Panthera leo* [31]; pumas, *Puma concolor* [32, 33]; snow leopards [9]; Cheetah, *Acinonyx jubatus* [34]), but are poorly known for jaguars. The most recent viability analyses conducted for jaguar populations used life history data inferred or derived primarily from captive jaguars and other wild felid species [20, 35, 36, 37]. Life history parameters of jaguars have been estimated only from a very low density population in the northern-most part of their range, the semi-arid desert scrub environment of Sonora [10]. Although this represents an interesting case study, it is not representative of the majority of the global jaguar population, which inhabits moist tropical forest [37]. As such, there is a need for reliable estimates of life history parameters from jaguar populations across their range.

Single ‘snap-shot’ surveys to assess jaguar population status (abundance or density) are common in the literature, often conducted as baseline assessments without the financial and/or logistic capacity to repeat surveys over time or across the wider landscape. The estimates are often biased or imprecise due to methodological inadequacies associated with the size of camera grids, camera placement and low detection probabilities (see [3, 38, 39, 40]); however they are frequently presented as a one-time static but true representation of the number of jaguars in an area, with no method of validation (cf. [9], who validated snow leopard abundance estimates from camera trap data with data from known radio-collared individuals in the study area). It is therefore of value to consider how robust single survey population estimates are for jaguars, especially for survey designs that are logistically and financially realistic. For low density, wide-ranging and relatively long-lived species with complex social dynamics such as jaguars, we may expect a high variation among abundance estimates from repeated surveys if they are constrained in space or time, as animals become temporarily unavailable for detection when they move off the trapping grid. Interpreting one-off abundance estimates and detecting true population change at the local level (i.e. within the area of a standard camera grid) from a time series of abundance estimates, requires an understanding of the local population structure and the ability to distinguish between sexes and between residents and transients. Only by monitoring individuals over multiple years, and understanding the population structure, is it possible to retrospectively interpret an abundance estimate for any given year. For example, in Chitwan, Nepal, the tiger population monitored over 7 years was found to have high variance among years in abundance estimates, which the authors attributed to a stable layer of breeding residents and a fluctuating layer of non-breeders [41]. In contrast, in South Gobi, Mongolia, the snow leopard population monitored for 4 years was found to have low variance among years in abundance estimates, but high turnover of individuals and associated shifts in sex ratio [9]. It is therefore useful to assess robustness of abundance estimates by explaining the variance among years in terms of local population structure.

In this study, we investigated the demography and dynamics of a jaguar population in the Cockscomb Basin Wildlife Sanctuary in Belize, over a 14-year period. The basin is a well-protected tropical forest system, impacted minimally by anthropogenic activities, and recognised for supporting a relatively high density of individuals compared to other areas within the jaguar range [3, 42, 43, 44]. Water is plentiful year-round due to a dense network of waterways throughout the site, and field data suggest that populations of the main prey species have remained stable or increased since the area was first protected in 1986 [45, 46]. Cockscomb Basin is at the eastern outer edge of, and contiguous with, the Maya Mountain Massif, approximately 5,900 km^2^ of national protected forest. As such, we expected high survival and a stable population of long-lived residents, which would provide an ideal opportunity to estimate life history parameters and to assess the validity of single ‘snap-shot’ abundance estimates. We investigated the variation between closed population abundance estimates derived from a standardised camera-trap survey repeated every year and assessed the population stability by quantifying the underlying structure in terms of sex ratio, residency status and longevity. Following [8, 10], we used a robust design, open population model to estimate apparent survival, recruitment and temporary emigration rate of the population. This is the first long-term jaguar study with sufficiently large samples to quantify population dynamics and demographic change.

## Methods

This study was carried out using non-invasive methods of monitoring mammals. We obtained permission from the Belize Audubon Society and the Belize Forest Department to carry out this research.

**Fig 1:**
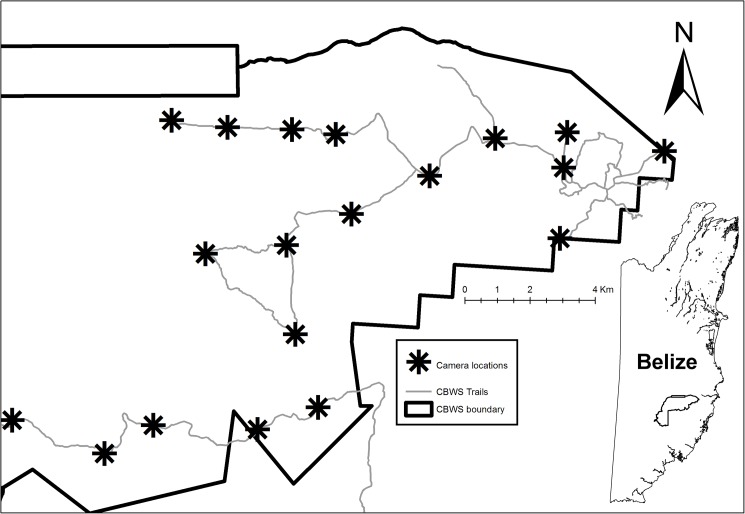
The 19 permanent camera station locations within the Cockscomb Basin Wildlife Sanctuary (CBWS). Map of CBWS showing the camera locations, the trail system, and an inset of the National map of Belize, indicating the location of CBWS.

The study was conducted in Cockscomb Basin Wildlife Sanctuary (here-on, CBWS or sanctuary), 490 km^2^ of moist broad-leaved tropical forest. The area was selectively logged until 1981, and protected in 1986, and is now a mosaic of regenerating secondary forest in several stages of succession. Many of the old logging roads in the eastern part of CBWS are maintained as tourist trails or patrol routes (Fig 1), providing easy, and presumably, preferred travel routes for jaguars to move through the dense secondary vegetation [46, 47]. Relative to other tropical moist broad-leaved sites, the Cockscomb Basin supports a high density of jaguars [43].

Nineteen locations, with two cameras per station, were used annually to survey during the February-June dry season from 2002 to 2008, and from 2011 to 2015, covering an area of ~100 km^2^ (Fig 1). Neighbouring stations were separated by 1.07 to 3.05 km (mean = 2.02 km); while the furthest distance between stations at the extremities was 21.6 km. Survey duration ranged from 59 to 98 days, except for the 2013 survey which was conducted for a full 365 days to explore seasonal variation in detection probability. The camera distribution continued the original deployment of [42, 43] as optimal locations with high capture probability. The configuration followed the original protocol of [6] and was maintained for consistency and comparability. Some stations were kept in use pre- and/or post-survey period for continued monitoring of presence of individual jaguars in specific areas. Additionally, seven short-term small-scale surveys of 32–47 days, each within 30–70 km^2^, were conducted with high densities of cameras (3–5 cameras per 10 km^2^) within the main survey grid in 2003, 2004, and 2005 [43, 48, 49]. Data from these ‘nested surveys’ contributed to the estimation of jaguar ages, and to evidence of the presence of individuals within the study area outside of the main survey periods. From 2003–2008, we used traditional film camera traps (CamTrakker, Cuddeback and DeerCam) with an enforced 3-minute delay between exposures to prevent excessive photos of herding species such as peccaries (*Tayassu sp*.). From 2011 onwards, we used digital camera traps (Pantheracam) with a minimum delay of 8 seconds between successive photo triggers. Every photograph was stamped with the time and date. We identified individual jaguars based on their unique spot patterns, and assigned sex based on the presence or absence of testicles, following [50].

### Open population model

We used the robust design model in program MARK [51, 52] to estimate probabilities of detection, survival, temporary emigration, and population abundance as a derived parameter. We used the 12 dry season surveys (2002 to 2008, and 2011 to 2015), as the primary occasions, accounting for the missing years in the model design; and week-long periods as the secondary occasions for the closed population estimates within the robust design (S1 File). As the 2013 survey ran for a full year, we only used a subsample of 100 days from the dry season period. We generated 20 *a priori* models, allowing the parameters to be constant or variable from one survey year to the next, or to differ by sex. We started with a general model with full time and sex dependency in all parameters. We included models in which temporary emigration in the primary periods (ɣʹ = probability of remaining outside the sample and ɣʺ = probability of temporary emigration) was dependent on temporary emigration status in the previous period (Markovian movement) and models in which it was not (random movement); and included a no emigration model where ɣʹ = 1 and ɣ ʺ = 0 [51]. For the detection parameters (capture probability, p, and recapture probability, c), we set p = c in all models for comparison with other studies; and allowed it to vary with the type of camera trap (film or digital), time, and sex. We included the camera/ time model following [8, 10], the only other long-term camera-trap study on jaguars, which found that detection parameters differed between sessions that used film versus digital camera traps. We used Akaike's Information Criteria (AIC) to select the best-fitting models for apparent survival, and temporary emigration, and population abundance (for details on the analysis, see [8]). Following [8], we defined apparent survival as remaining alive within the study area (not dying or permanently emigrating). We calculated the finite rate of change in abundance between each of the primary sampling periods (λ) as N^t+1N^t, where $\hat{N}$ is the abundance estimate at time t. We then calculated geometric mean annual rate of change, following [8] and the associated variance following [53]. We calculated the annual number of new recruits into the local population as $N_{t+1}-{\hat{N}}_{t}{\hat{\varphi }}_{t}$, where ${\hat{\varphi }}_{t}$ is apparent survival at time t, following [8].

### Detection frequency

To further investigate variation in the detection of individuals, we recorded detection/non-detection and detection rates per survey for each individual jaguar. We tested for a difference between: (1) the number of male and female individuals detected each year and (2) mean detection rate of males and females each survey year, using Wilcoxon paired tests. To ensure independence, we only included consecutive detections that were ≥ 24 hours apart, per individual We further used the 2013 full-year survey to assess variation in detection probability for males and females throughout a year. We sub-sampled the year into four quarters of 3 months each (equivalent length to a regular survey period) and assessed the number of individuals detected and frequency of detection in each quarter.

Detection of individual jaguars may differ among years, with individuals being detected in some survey years and not others. We investigated whether the detection/non-detection of individuals across years differs between males and females, by regressing the total lifespan (y) in the study area (last year of detection minus first year of detection) against the number of years an individual was actually detected in the study area (x). If the slope m, within the equation of y = m x, approximates 1, individuals were consistently detected every year. If slope m > 1, individuals would tend to skip years within their total lifetime detection record. We compared the regression slopes of males and females to determine whether there was a difference between the sexes in the consistency of capture over the years.

We separated individuals into ‘residents’ and ‘transients’; a local resident was classified as having been detected within the survey grid for at least three consecutive years, while local transients were the remaining group, being detected ≤ 2 years in a row, with either few years of detection in total or irregular detections throughout the study period. Our definition of residents was based on the assumption that individuals who were detected for at least three consecutive years had the core of their ranges within the survey grid. Thus the terms resident and transient apply to the study area only. As such, a transient could be a true resident outside of the survey grid; while residents hold tenure within the survey grid, and potentially beyond its boundaries. We used the period 2003 to 2007 as the longest running period of consecutive survey years, using data from 2002 and 2008 to be certain of local residency status for the period 2003–2007. As sample sizes were not sufficient for females, we limited the analysis to males. We tested our definitions of residency by comparing the number of years of detection and minimum age (number of years between first and last detection) between the two groups, using a permutation test with Monte Carlo function [54]. Compared to local transients, we expected local residents to have a greater proportion of detection years for their age, a longer tenure within the study area, and more detections.

### Age structure

For each year, we calculated the minimum age of the individuals detected, using their year of first and last detection. We assumed that adults were at least 2 years old on first solo detection, as young jaguars usually associate with their mother for approximately the first 24 months of life [55].

All statistical analyses were performed in R [56].

## Results

One hundred and five adult jaguars were detected from 2002 to 2015 in 3,075 detection events; 57 males, 31 females and 17 individuals of unknown sex (2720; 332; and 23 detections, respectively). Per year, significantly fewer females were detected than males (paired Wilcoxon Test: V = 45, *p* < 0.01, n = 10 survey years; Median males = 18 individuals [15–19], Median females = 5 individuals [4–6]). Four cubs were detected for the entire study period and these were of advanced age (> 6 months).

### Open population models

The best-fitting model indicated that survival and temporary emigration were constant across years and equal between the sexes, while the detection probability varied between the sexes and with camera type (Table 1). Even though the top six models showed variation in terms of constancy or variability across time or sex, the estimated parameter values were very similar for all six models. We therefore present the model averaged parameter values as a good overview of estimated parameter values (Table 2). Both sexes had a high survival probability of ~0.78 from one year to the next, i.e., the probability that an individual would permanently leave the population (die or permanently emigrate) from one year to the next was ~0.22. Additionally, the probability of temporarily leaving the study area and returning later was ~0.20 (Table 2). Detection probability for males was 2.1 to 9.4 times greater than for females (Table 2). Detection probability was constant through time for males and females for the period 2002–2008 when film cameras were in use, but varied among years for the period when digital cameras were used 2011–2015. Comparing the detection probabilities estimated during the film and digital periods, male detection in the digital period ranged from 0.75 to 1.3x the detection during the film period, and for females detection in the digital period ranged from 0.71 to 2.3x that of film period.

**Table 1:** Model selection for capture-recapture camera-trap data for jaguars, using robust design population models, CBWS 2002–2015 (years 2009–2010 missing).

Model	AICc	Δ AICc	AICc Weights	Model Likelihood
φ (.), ɣʹ = ɣʺ(.), p = c (sex*camera_(./time)_)	3351.69	0.00	0.70	1.00
φ (sex), ɣʹ = ɣʺ(.), p = c (sex*camera_(./time)_)	3353.77	2.08	0.25	0.35
φ (time), ɣʹ = ɣʺ(.), p = c (sex*camera_(./time)_)	3358.19	6.50	0.03	0.04
φ (sex_(time/.)_), ɣʹ = ɣʺ(.), p = c (sex*camera_(./time)_)	3359.04	7.35	0.02	0.03
φ (sex_(./time)_), ɣʹ = ɣʺ (.), p = c (sex*camera_(./time)_)	3364.13	12.44	< 0.01	0.00
φ (sex*time),ɣʹ = ɣʺ(.), p = c (sex*camera_(./time)_)	3369.86	18.17	< 0.01	0.00
φ (sex*time),ɣʹ = ɣʺ(.), p = c (sex*camera)	3381.88	30.19	0	0.00

Seven best-fitting models ranked in order of best fit, using Akaike's Information Criteria (AIC).

φ apparent survival

ɣʹ = ɣʺ: temporal immigration and emigration are random i.e. do not depend on the location during the previous year

p = c capture and recapture probabilities are equal

(.) null model, no time or sex dependence

(sex) sex dependence i.e. varies between sexes

(camera): camera dependence i.e. varies between camera type (film or digital)

(camera_(./time)_): p and c are constant in years with film cameras (2002–2008) and variable among years with digital cameras (2011–2015)

(time) time dependence (varies between years)

(sex_(time/.)_) male time dependence i.e. variation between years for males

(sex_(./time)_) female time dependence i.e. variation between years for females

**Table 2:** Jaguar survival, detection, immigration and emigration probabilities for the jaguar capture-recapture camera-trap data from CBWS, 2002–2015 (years 2009–2010 missing).

Year	Survival ^a^		Detection probability		Abundance		Temporary Emigration
	Male	Female	Male	Female	Male	Female	Male/Female
2002	0.79 ± 0.05	0.79 ± 0.06	0.36 ± 0.02	0.07 ± 0.02	11.31 ± 0.57	NE	0.21 ± 0.04
2003	0.79 ± 0.05	0.79 ± 0.06	0.36 ± 0.02	0.07 ± 0.02	9.16 ± 0.41	4.34 ± 2.44	
2004	0.78 ± 0.05	0.78 ± 0.05	0.36 ± 0.02	0.07 ± 0.02	14.16 ± 0.41	12.09 ± 4.30	
2005	0.79 ± 0.04	0.79 ± 0.05	0.36 ± 0.02	0.07 ± 0.02	15.11 ± 0.33	3.77 ± 1.98	
2006	0.79 ± 0.04	0.78 ± 0.05	0.36 ± 0.02	0.07 ± 0.02	16.29 ± 0.55	2.17 ± 1.66	
2007	0.77 ± 0.06	0.78 ± 0.06	0.36 ± 0.02	0.07 ± 0.02	19.22 ± 0.47	12.09 ± 4.30	
2008	0.79 ± 0.04	0.79 ± 0.04	0.36 ± 0.02	0.07 ± 0.02	11.20 ± 0.45	6.51 ± 3.09	
2011	0.78 ± 0.05	0.78 ± 0.05	0.47 ± 0.03	0.05 ± 0.03	18.01 ± 0.07	8.65 ± 4.96	
2012	0.77 ± 0.06	0.78 ± 0.06	0.27 ± 0.03	0.06 ± 0.03	17.14 ± 0.39	7.91 ± 2.96	
2013	0.78 ± 0.05	0.78 ± 0.04	0.40 ± 0.03	0.12 ± 0.04	14.01 ± 0.09	4.70 ± 1.10	
2014	0.78 ± 0.05	0.78 ± 0.05	0.33 ± 0.04	0.16 ± 0.04	12.06 ± 0.26	7.81 ± 1.10	
2015	NE	NE	0.37 ± 0.04	0.16 ± 0.04	15.15 ± 0.40	9.60 ± 1.72	

Estimates presented correspond to the model average ± standard error across the top six models for all parameters obtained, using the robust design models.

NE: not estimable. No data available for 2016 so survival cannot be estimated for 2015–2016.

^a^ Survival measure is apparent survival from the year of the row to the next. We consider apparent survival rather than true survival due to the absence of dead recoveries

**Fig 2:**
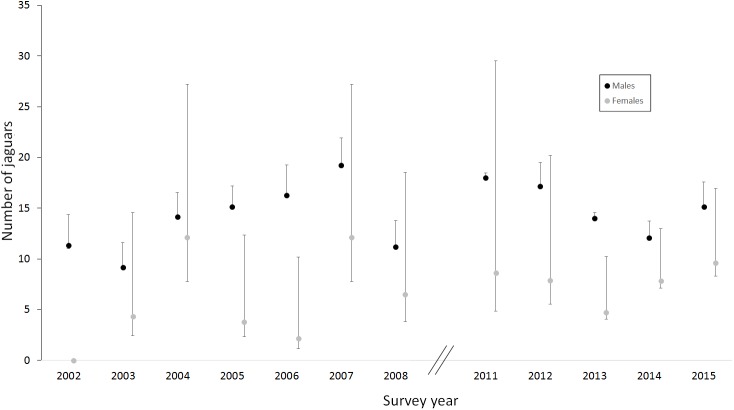
Yearly abundance estimates for male and female jaguars in the CBWS study area. Average abundance with confidence intervals per survey year (2002–2015), using robust design models in program MARK. Data were missing for the years 2009 and 2010

Female abundance estimates were relatively imprecise compared to those for males due to the lower numbers detected and lower detection (capture and recapture) probability of females (Fig 2 and Table 2). Although the mean abundance estimates for females were lower than for males, the 95% confidence intervals generally overlapped within years between the sexes, except for four years when the female estimates were lower than for males (2002, 2005, 2006 and 2013, Fig 2). Estimates of male abundance varied among the years, without showing any clear trend (Fig 2). Our estimates of male and female abundances differed by 10 individuals among the years (male abundance 9–19, female abundance 2–12), with more precise and higher estimates for males presumably due to their higher detectability (Fig 2).

Based on the derived abundance estimates, the annual population change (λ) varied from positive to negative among the years, and between the sexes (Table 3). Over the 14-year period the mean annual rate of abundance change of males was 0.98 (var = 0.07), while for females it was 1.06 (var = 0.76). Overall, the mean annual population change was positive (1.04, var = 0.17). The number of new recruits into the population varied from year to year and between the sexes. On average, three new recruits of each sex entered the population each year (male mean = 3.3, range 0 to 7 individuals; female mean = 2.8, range 0 to 10 individuals; n = 10). Overall, the average number of new jaguar recruits was 6.1/year (var = 36.0).

**Table 3:** Annual change and recruitment of jaguars in the CBWS study area.

Year	Population change (λ)			New recruits		
	Male	Females	Total	Male	Female	Total
2002	0.81	-	1.19	0.2	0.0	0.2
2003	1.55	2.79	1.94	6.9	8.7	15.6
2004	1.07	0.31	0.72	4.1	0.0	4.1
2005	1.08	0.58	0.98	4.4	0.0	4.4
2006	1.18	5.57	1.70	6.4	10.4	16.7
2007	0.58	0.54	0.57	0.0	0.0	0.0
2008	-	-	-	-	-	-
2009	-	-	-	-	-	-
2010	-	-	-	-	-	-
2011	0.95	0.91	0.94	3.1	1.2	4.3
2012	0.82	0.59	0.75	0.8	0.0	0.8
2013	0.86	1.66	1.06	1.1	4.1	5.3
2014	1.26	1.23	1.25	5.7	3.5	9.3
**Mean**^**a**^	**0.98**	**1.06**	**1.04**	**3.3**	**2.8**	**6.1**
**Var**	**0.07**	**0.76**	**0.17**	**6.8**	**15.1**	**36.0**

Annual change in abundance and new recruits into the study area, 2002–2014; no females were detected in 2002, and no data were collected in 2009 or 2010.

^a^ Geometric mean for λ

### Detection frequency

The mean detection rates of individuals per survey year were significantly lower for females (paired Wilcoxon Test: V = 45, *p* < 0.01, n = 10 years; Median males = 14 detections/individual/ survey year [11–15], Median females = 3 detections/individual/survey year [3–4]). During the 365-day survey (2013), we detected 12 females (76 detections) and 17 males (514 detections), revealing a less-skewed sex ratio than the 3-month surveys. Sub-sampling the 12-month survey period into 3-month intervals revealed that only 42%, 33%, 58% and 50% of the 12 females were detected in the 1^st^, 2^nd^, 3^rd^ and 4^th^ quarter, respectively, compared to 76%, 82%, 88% and 82% of the 17 males.

**Fig 3:**
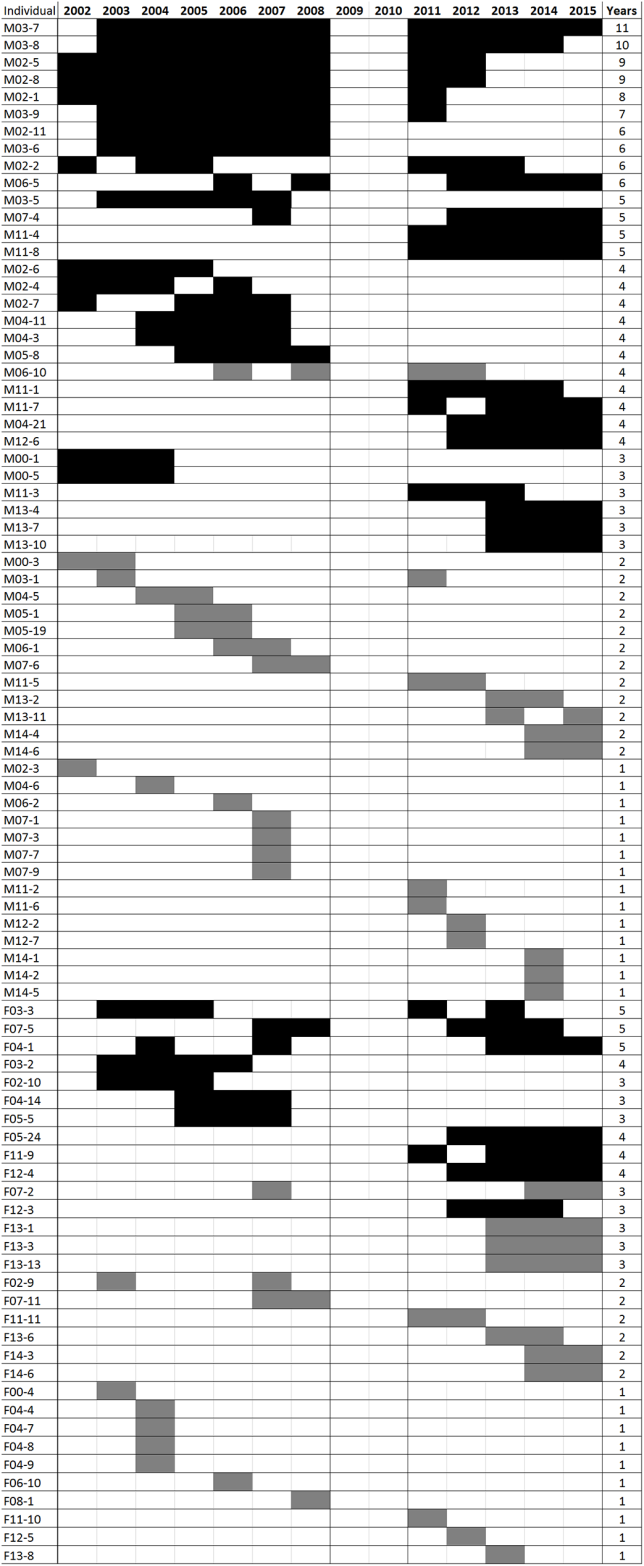
Presence per year for all adult male and female jaguars detected in the study area between 2002 and 2015 (no data for 2009 and 2010). Individuals are ordered by number of years of detection. Black bars indicate individuals detected for ≥3 consecutive years (‘residents’ of the survey area), dark grey bars represent individuals detected for ≤ 3 consecutive years (‘transients’ of the survey area).

**Fig 4:**
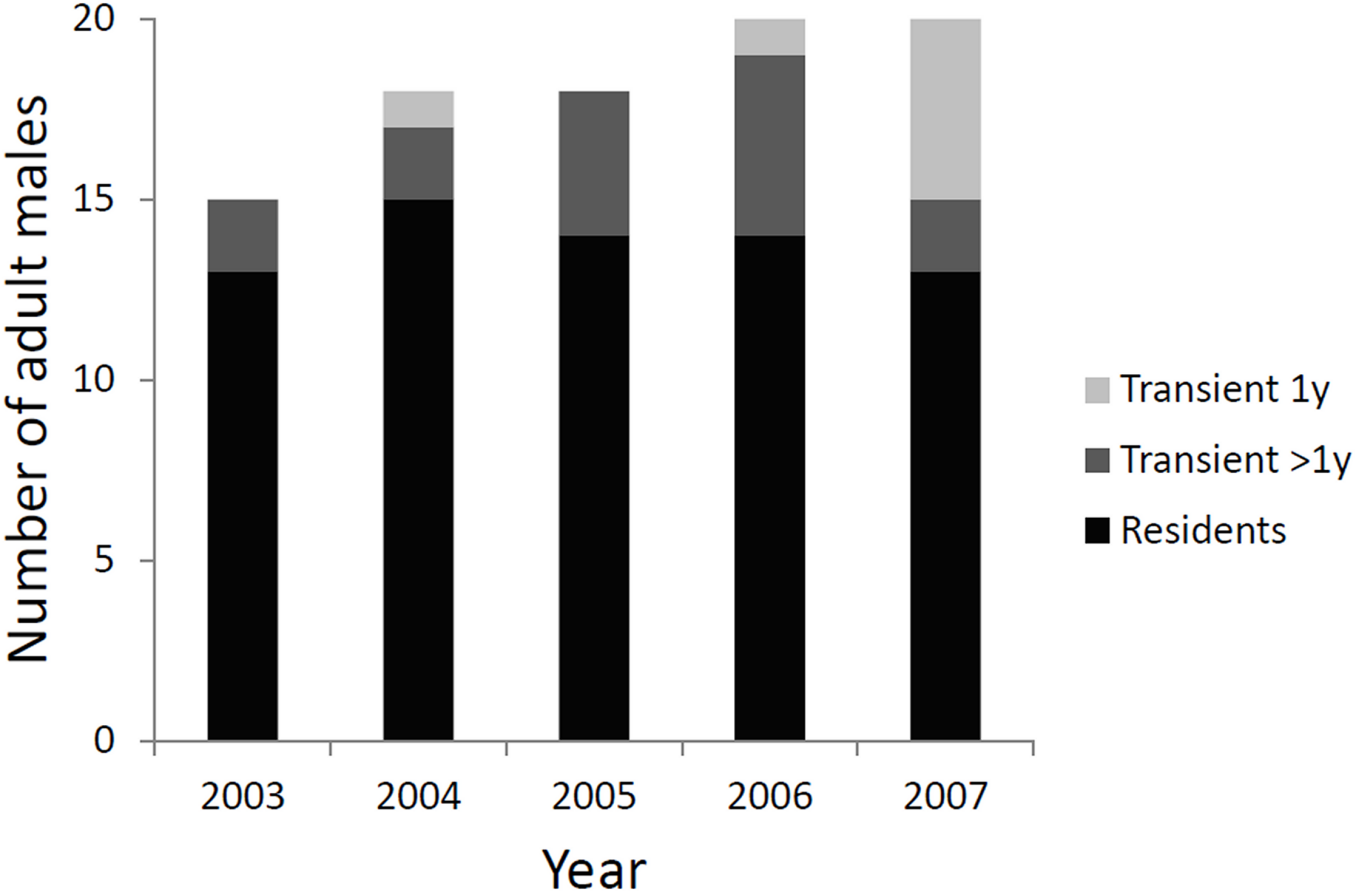
Number of male jaguars detected each survey (2003–2007), separated into ‘residents’ and ‘transients’. Residents were defined as detected ≥ 3 consecutive years (black). We separated transients into two parts, 2 consecutive years of detection (dark grey), 1 year of detection (light grey).

We found a significant linear relationship between male detection frequency and the time interval between first and last detection (F = 71.5, df = 34, *p* < 0.01, adjusted R^2^ = 0.67, y = 1.2 x—0.2), indicating that an average male tended to be detected in consecutive years. We also detected a significant linear relationship for females (F = 16.9, df = 16, *p* < 0.01, adjusted R^2^ = 0.48, y = 1.9 x—1.2), however the slope of ~2 indicates that an average female would be detected every other year. Close inspection of the raw data showed that annual detections for females were sporadic, while the majority of males were detected for at least three consecutive years, and assigned ‘local resident’ status (Fig 3). The number of local male residents remained relatively stable across years (12–15 individuals), while the number of local male transients fluctuated from 2 to 7 individuals per year (Fig 4).

Residents had a significantly longer tenure within the study area than did transients (permutation test, mean time between 1^st^ and last detections, resident = 5.5 ± 2.9 years, transient = 2.7 ± 3.5 years, *p* < 0.05) and were detected in significantly more years than transients (permutation test, resident = 5.7 ± 2.1 years, transient = 2.2 ± 1.2 years, *p* < 0.01). Residents were more active on trails than transients (detections per location per survey-year 2003–2007, for residents: median ± IQR = 1.7 ± 0.7, *n* = 17; transients: median ± IQR 1.0 ± 0.8, *n* = 10; two-tailed Mann Whitney test, *W* = 125.5, *p* < 0.05).

### Age structure

**Fig 5:**
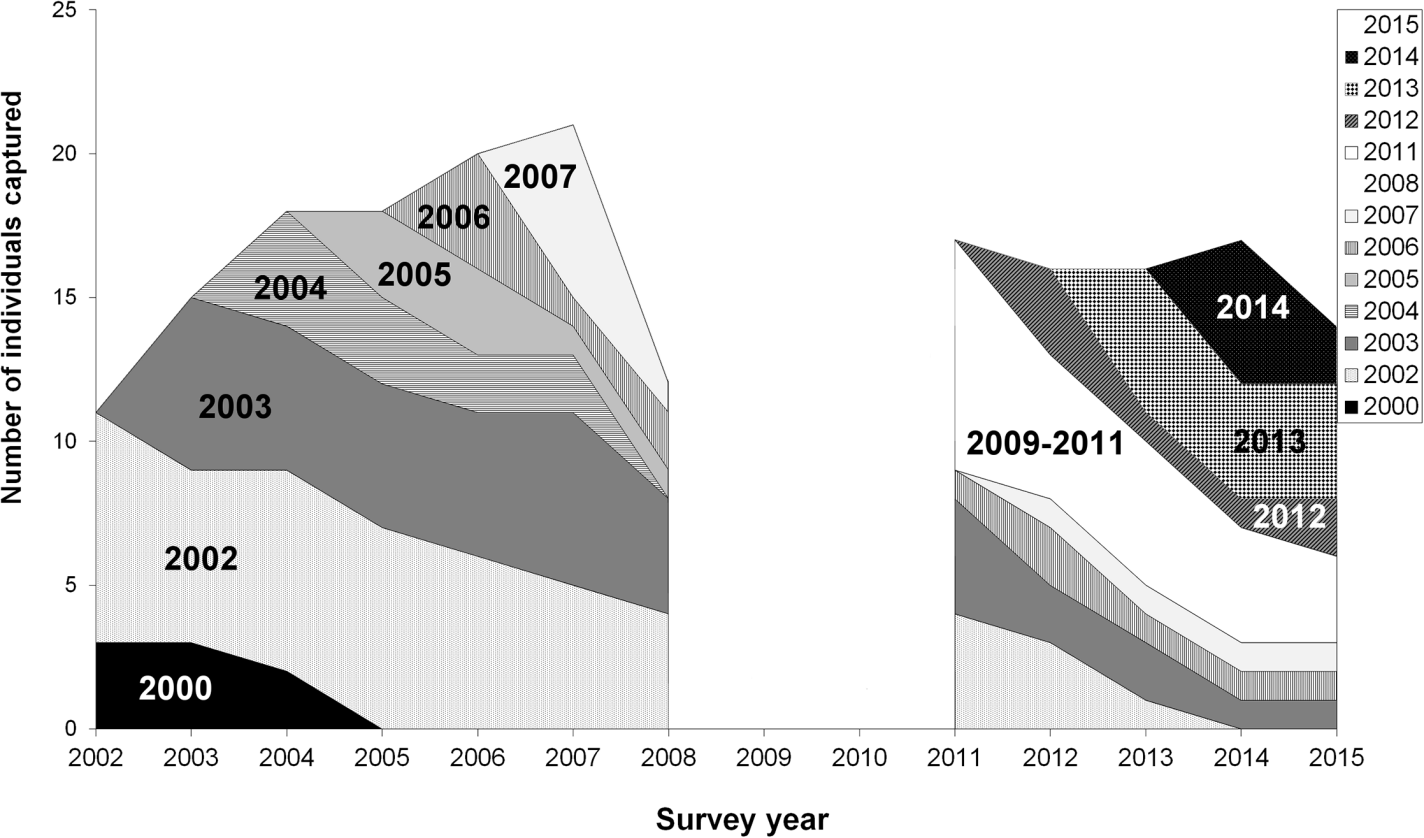
Number of adult male jaguars detected each year, separated into cohorts according to year of first detection. Yearly cohorts of jaguars represented with different colour bars (see legend in figure). Note that there were no new male recruits in the year 2008 and individuals first captured in 2011 (white cohort, labelled 2009/2011) may have entered the study area as early as 2009 or 2010, but remained undetected as surveys were not conducted in these years.

Our records indicated a conservative maximum age of at least 14 years for males and 13 years for females. Almost half of the known males were consistently detected in the study area, with 48% detected in ≥ 3 consecutive years (Figs 3 and 4). Males first detected in 2002 or 2003 comprised 52 to 67% of the males detected each year from 2004 to 2008 (Fig 5). In 2011, at least half of the males in the sample were > 10 years old. In 2013, 21% of the males present were first detected in 2002 or 2003, and by 2015 this was reduced to 7%, representing a single surviving individual (Fig 5). This was comparable to our model estimate of adult survival (φ) of 0.78, which equates to 7% of young (2-year old) adults reaching 13 years of age (0.78^11^ = 0.07). The overall pattern is one of low recruitment of new males into the survey grid per year, and a high retention of males in the area, as reflected in the open population model. Because the detection frequency of females was low and individuals were not detected every year (Fig 3), it was not possible to describe age structure for this sex.

## Discussion

Through long-term monitoring of jaguars at a single site, we assessed jaguar survival and documented aspects of jaguar life history that are otherwise difficult to observe. We combined data from ‘snap-shot’ surveys (3 months each) repeated over 14 years, with a single year-round survey (12 months) to estimate population parameters, better understand jaguar life history, and to assess the validity of single ‘snap-shot’ abundance estimates. To our knowledge, this is the longest-monitored jaguar population to date in Central America, closely followed by the nearby population of the Mountain Pine Ridge, Belize with equal number of continuous years [57]. Because the study area experiences minimal human impact, populations of the main prey species have remained stable and/or increased since the area came under protection [45], therefore we expected high jaguar survival. Apart from some of the known ≥ 12 year olds, nearly all detected jaguar individuals displayed good body condition (no evidence of protruding hips or spine), suggesting that the population is not limited by prey availability. Apparent survival of jaguars in the study area was high (φ = 0.78), similar to estimates for other large felid species in protected areas (leopards in Kruger National Park, South Africa 0.82 [28]; tigers in Nagarahole, India 0.77; [8]; male Siberian tigers in Sikhote-Alin Biosphere Zapovednik, Russia 0.75 [26]; snow leopards in Tost Mountains, Mongolia 0.83 [9]). These survival estimates exceed that of the low density jaguar population in Sonora, northern Mexico (survival = 0.50 to 0.55, [10]), which is more similar to survival estimates for large felid populations experiencing with human-induced mortality and negative growth (e.g. male pumas North Pacific United States 0.55, [33]), male leopards, Phinda, South Africa (0.45, [29]). The CBWS jaguar population acquired on average six new recruits each year, ranging from 0 to 17; this is equivalent to a mean annual increase of approximately 4%, which is comparable to the 3% increase detected for tigers in Nagarahole, India [8]. Our conservative estimate of the maximum lifespan of male jaguars in the wild (at least 14 years) is the first published to date, and is similar to estimates for males of other large wild felids of the *Panthera* genus in well protected areas (e.g. lions (*P*. *leo*), rarely >12 years; tigers,12 years; leopards (*P*. *pardus*), 14 years; [58]. At least 35% of the known male residents reached ≥ 10 years. We conclude that true survival of individuals remaining in CBWS is higher than the apparent survival of 0.78, which includes dispersers that never returned.

The male-biased sex ratio consistently observed during each primary sampling period does not reflect the demographic structure of the population, but rather is likely an artefact of the lower detectability of females than males when using trail-based cameras. This is confirmed by the fact that we detected more females, and thus a more even sex ratio, when we monitored the population continuously for a year, compared to 3-month survey periods; however the detection probability of female individuals remained low with few detection events per female [59].

The lower detectability of females versus males can explained in terms of behavioural differences that influence trail use. We observed an extremely low detection rate of adult females with cubs (only 4 out of 332 detections over 14 years, equivalent to 1.2% of female detections), and relatively sporadic detection of lone females from year to year on the trail system. This suggests that when females are denning and/or have vulnerable dependents, they likely avoid areas with high male traffic, potentially returning intermittently to advertise their presence on the trail system when young have dispersed and they are ready to mate (see also [60]). Such behaviour may be expected in this study area due to the extensive male-male range overlap [46, 48], which may provoke infanticide, as is common in other large cats (e.g. [32, 61, 62, 63, 64]).

Our models suggest that temporary emigration from the camera grid was random, and occurred with an estimated probability of 0.20 from year to year for both sexes. Thus, during each primary sampling period, 20% of the sampled jaguars were unavailable for detection via camera traps [8]. For females, we assume that they remained within the survey area but temporarily avoided the trail system (and our cameras), according to their reproductive status [47, 58]. For males, temporary emigration may be an consequence of an insufficient camera grid size relative to home range size such that some sampled individuals moved beyond the grid boundaries, becoming temporarily unavailable for detection (edge effects). We expect that this is more likely for males than females, as male jaguars in this region have larger ranges than females, with upper estimates exceeding our study area size [35, 65].

Male abundance estimates varied two-fold among the years, while those of females varied up to six-fold. We obtained more precise estimates for males than females due to the greater detectability of males. High temporal variation in detections at point locations, and thus estimates of local abundance, should be expected for wide-ranging, low density species such as jaguars that have complex socio-spatial dynamics. Local detections (and thus estimates of local abundance) may increase or decrease, for example, when a female contracts her range for denning, multiple males converge on a female in oestrus, individuals migrate following prey, or young adults disperse.

For the females in our study, the high variation in abundance estimates among survey years may be explained in terms of the sampled females becoming temporarily unavailable for detection on the trail system when they are denning or with dependents, and available for detection when in heat. This cycle may span up to 18–24 months, until dependents disperse. Thus the cycle of detectability on the trail system will exceed the three month primary sampling period, resulting in sporadic detection of individuals and high variation in estimates of local abundance between the years.

Although our estimates of male abundance fluctuated among survey years, we identified a stable population of residents and a fluctuating population of transients, as has been observed in tigers and pumas [41, 55]. Our definition of local residents refers to individuals that were detected on the trail system for three consecutive years or more. Although these individuals may have ranged beyond the grid boundaries, we identified them as ‘local’ residents because the cores of their ranges likely lay within the survey grid. This definition is supported by our finding that this subset of individuals had longer total tenure in the survey grid than other males; and, in any given year, they were more frequently detected walking the trail system. The transients could represent various demographic/social groups: young dispersing adults, nomads, subordinate individuals that avoid the trail system, or individuals whose home ranges are mainly outside of the survey area and occasionally overlap with the edge of the camera grid.

Our results support the hypothesis of a thriving stable jaguar population in the CBWS, with high survival and long tenure for residents, at least for males. However the variance among abundance estimates would suggest a fluctuating population. We interpret this as local demographic change or idiosyncrasies of each survey period and not representative of the wider population processes within the region. Although the number of individuals detected during our primary sampling periods can be considered high for jaguar studies (11–24 individuals) [3, 40], we have shown that if the sampling periods are treated as single, one-off, snap shot surveys they are not sufficient to include a representative sample of the total demographic variation of a population for a meaningful abundance estimate. Increasingly, one-off population estimates are being used to extrapolate to the landscape, biome, national, regional and global levels (e.g. [37, 66, 67, 68, 69]). In the absence of repeated population estimates from the same sites, or an understanding of the population structure at the sites, such extrapolations should interpreted cautiously. Large-scale camera surveys that cover a sufficient area, and at sufficient camera density, to detect a representative sample of demographic variation in the target population within a single snap-shot survey are generally rare to non-existent. For jaguars, we do not know how large such an area should be, but this could potentially be enormous and financially and logistically impossible to survey. In a review of 72 studies that estimated jaguar density from camera-trap data, [40] reported that only nine of the studies deployed camera traps across an area equivalent in size to at least one average male home range. Simulation studies in [40] showed that camera grids should cover a minimum of a single average home range size, which we roughly accomplish within our survey [35, 46, 65]. However the simulated data in [40] are based on SECR generated home ranges, concerning independently placed activity centres surrounded by average sized elliptically shaped home ranges. Although informative, this does not reflect anything close to the potential heterogeneous ranging behaviour of jaguars across space and time in a population. If cameras cannot be deployed across a wide enough area to detect a representative demographic sample of the target population, we recommend repeating surveys over multiple years, and combining robust design population models with quantification of tenure. As we have demonstrated, in this way it is possible to account for the local fluctuations associated with social processes (e.g., denning, courtship, avoidance of antagonistic interactions, and the exploration or take-over of new ranges) when assessing the population size and stability.

By sampling for 12 months continuously, we detected individuals with otherwise low or zero detection probabilities on trails during 3-month snap-shot survey periods. Therefore, we recommend extending the length of the ‘standard’ survey period (3 months) in areas where detection rates are very low in order to better understand the population structure within the study area. This will equally allow subsampling of the yearlong capture history into multiple short term segments for closed population capture-recapture analyses. Multiple estimates per year allow quantification of short term fluctuation in abundance/density estimates and study of ideal time period for satisfaction of the closure assumption.

As with [10], our study demonstrated that detection probability varied with the functionality of the camera trap. This is not unexpected: compared to modern digital models, the old-fashioned film camera traps are known for having slower sensors and higher failure rates compared to our Panthera cameras from the latter part. With the reduced cost and increased energy efficiency of more modern camera traps, long-term deployment will become more cost effective, requiring fewer battery and maintenance checks. Ideally, camera stations will be deployed permanently, allowing researchers to subsample camera data up to whatever level a question demands. This may range from following individuals through time to estimating population-level parameters and tracking population dynamics.

To estimate survival and monitor population change, repeated surveys over multiple years are necessary; or better still, continuous monitoring over multiple years. Where jaguars live in a protected population, adults of at least 10 years old are common, and a lifespan of 14 years is our current conservative estimate of longevity. Therefore we recommend monitoring programs of at least 5–10 years, to track the dynamics of half, or ideally, an entire cohort. Beyond population dynamics, long-term monitoring accrues additional insights into life history providing a more comprehensive picture of a jaguar population; and allows us to validate, or at least understand, the underlying dynamics that contribute to abundance estimates based on single ‘snap shot’ surveys.

The individual recognition of jaguars from their spot pattern allows us to follow individuals through time without the need for invasive capture and marking. Five of the seven large felid species have such individually unique pelt patterns, and a diversity of medium and small-sized carnivores are also camouflaged with spots, rosettes, or stripes, or other unique markings, allowing for camera-based monitoring of otherwise elusive species. We advocate the use of camera traps for long-monitoring of such populations, and encourage granting bodies to recognise the multiple benefits of funding long-term monitoring programs [11].

## Supporting information

S1 File (XLSX)
